# Knowledge, attitude, and perception of oral and maxillofacial surgery specialty amongst healthcare professionals, and the General Public from a Gulf Cooperation Council (GCC) Country

**DOI:** 10.1186/s12893-021-01064-y

**Published:** 2021-01-26

**Authors:** Mohammad Kamal, Mohammad Abdulwahab, Ahmed Al-Zaid

**Affiliations:** 1 Department of Surgical Sciences, Faculty of Dentistry, Health Sciences Center, Kuwait University, Safat, Kuwait; 2 Kuwait Dental Administration, Ministry of Health, Safat, Kuwait

**Keywords:** Perception, Awareness, Oral and maxillofacial surgery, Specialty

## Abstract

**Background:**

Oral and maxillofacial surgery specialty has grown rapidly in Kuwait in recent years. However, the general public and healthcare professionals remain unaware of its expanding scope of practice. The aim of the study is to assess public and professional (dental and medical) perception of the oral and maxillofacial surgical specialty in Kuwait.

**Methods:**

This is a cross-sectional study evaluating responses of dental professionals, medical professionals, and general public in Kuwait toward the oral and maxillofacial surgical specialty using a previously validated survey instrument with 100 participants in each group. Participants were asked to choose the most appropriate specialist to treat certain procedures across 4 disciplines: reconstruction, trauma, pathology, and cosmetic. Statistical comparison was conducted between dentists and medical doctors using Fisher’s exact test with a p-value of < 0.05.

**Results:**

Disparities were noted each group’s responses. Oral and maxillofacial surgery was preferred overall for most clinical scenarios in trauma (p < 0.001), pathology (p < 0.001), and reconstructive surgery (p < 0.001). Plastic surgery was preferred for cosmetic surgeries (p < 0.001).

**Conclusions:**

This study indicates the need to increase awareness especially towards cosmetic surgery procedures, and conduct health campaigns regarding oral and maxillofacial surgery among healthcare professionals, especially medical doctors, and the general public.

## Background

Oral and maxillofacial surgery (OMFS) is a relatively young surgical specialty that focuses on diagnosing and treating conditions in the head and neck region, acting as a bridge between dentistry and medicine [1, 2]. OMFS evolved significantly in recent years, triggering major technological and clinical advances in the fields of traumatology, dentofacial deformities, head and neck oncology and reconstruction, and temporomandibular joint disorders [3, 4]. Presently, OMFS offers a fairly broad spectrum of treatments, including distraction osteogenesis, implant surgery, tissue engineering, sleep apnea treatment, and esthetic facial surgery [3, 5, 6].

Despite being a well-recognized specialty of the facial skeleton by major hospitals internationally, disparities remain regarding proper recognition of OMFS’s scope and familiarity with OMFS surgical procedures among students, healthcare professionals, and laypersons [2, 4, 5]. Previous work in Kuwait by Haron et al. assessed the perception of OMFS by medical and dental professionals and found disparity regarding healthcare professional consultations for various conditions in the head and neck region based on a survey instrument evaluating 26 procedures [7]. A similar study in Saudi Arabia by Alnofaie et al. showed also significant differences between dentists and medical doctors perceiving OMFS [8].

The practice of OMFS in Kuwait officially began in the 1960s, mostly by foreign specialists visiting or practicing on regular basis. During later years, the development of the Kuwait healthcare and educational system led to local doctors being trained abroad in several North-American, European, Asian, and North-African countries to prepare them for the nationally subsidized Kuwaiti healthcare sector [7]. Over time, a heterogeneous group of OMFS surgeons formed with diverse training and a spectrum of clinical practice. The medical system in Kuwait is based on providing comprehensive care by the state-funded hospitals and many health care primary centers. Five health regions, with over 70 primary healthcare centers are responsible for managing the whole population in the state of Kuwait [9]. Primary healthcare centers are well staffed with general practitioners and general dentists who licensed thorough the Ministry of Health to offer mainly primary healthcare needs and serve as the initiating pathway toward referring cases to specialized centers for secondary and tertiary care services. The services provided across this multilateral system covers the whole spectrum of all medical and surgical specialties, along with the full spectrum of dental specialties. The Kuwait Ministry of Health attempts evaluate the standard of care, accreditation protocols, and the referral guidelines across all centers on regular basis [9]. There is currently insufficient reports on topics evaluating the medical referral system in Kuwait, with few studies published focusing mostly on patient safety culture and medical errors in the State of Kuwait [10, 11].

We shall investigate the current levels of knowledge, attitude, and perception towards the OMFS specialty in Kuwait among dental and medical professionals as well as the general public. This will help revisit our referral protocols, conduct needed awareness campaigns among the healthcare professionals to enhance their understanding of the scope of the specialty, and refine our medical and dental school’s curriculum to increase the OMFS educational content.

## Methods

### Participants

This cross-sectional survey drew one hundred subjects (n = 100) from both registered dentists and medical doctors working in all Kuwaiti healthcare sectors, the governmental and the private sectors, as well as the general public (laypersons) between April 1st to May 30th 2020. Participants were at least 18 years old and all participants voluntarily gave written consent to complete the questionnaire and were assured that their responses would be anonymous.

### Questionnaire

A previously validated and applied survey is used in the study with slight modifications by expanding the procedures list, and permission to use the questionnaire has been obtained from its authors [12, 13]. The questionnaire is divided into sections on demographics and general head and neck clinical conditions, some specific to OMFS practice (see Additional files, Questionnaire OMFS.pdf). Additional items are included in the survey to expand the spectrum and give a broader range of clinical scenarios. Each participant has 5 options consisting of 4 different specialists and an unspecified specialty. Participants are asked to indicate whom would be most appropriate or competent in treating each clinical condition, and only one option can be chosen for each condition. The questions are grouped and categorized by discipline (trauma, pathology, reconstruction, or cosmetic) and analyzed accordingly. Links leading to the online survey instrument was electronically to the medical and dental associations groups and to the public groups in Kuwait via e-mail, WhatsApp, Instagram, and Twitter pages. Ethical approval was obtained for this study from the Institutional Review Board (IRB) at Kuwait University Health Sciences Center.

### Statistical analysis

Statistical analysis is performed using SPSS Version 23.0 (IBM SPSS Statistics for Macintosh, IBM Corp., Armonk, NY, USA). Questionnaire responses from dentists and medical doctors are compared. Categorical data is compared using a chi-square test (cell count ≥ 5) or Fisher’s exact test (cell counts < 5). A p-value of < 0.05 is considered statistically significant given an 80% test power. Responses by laypeople are presented categorically and not included in the statistical comparison.

## Results

### Healthcare professionals (dentists and physicians)

The participants who completed the questionnaire consisted of 100 dentists and 100 physicians (Table 1). Participants were male (58%) and female (42%), with the majority aged 26–45 years. Medical doctors were older than their dentist counterparts (p = 0.012) and had more clinical experience (p = 0.033) with dentists possessing an average of 7.9 years (SD ± 7.4 years) compared to doctors holding 10.1 years (SD ± 6.9 years). Regarding receiving care, 27% had been supported by ear-nose-throat (ENT) specialists and 23% were treated by OMFS specialists, while 41% reported no prior medical treatment. Medical doctors had more personal experience with plastic surgery (PS) and ENT specialists while dentists had more personal experience with OMFS (p < 0.001).

**Table 1:** Respondent characteristics: number of participants, gender, age range, and years of experience

	Nr. participants	Gender		Age range—years					Years of experience
	n = %	Male	Female	18–25	26–35	36–45	46–55	> 55	
Dentists	100	62	38	17	52	22	9	0	7.9 (± 7.4)
Medical doctors	100	54	46	6	43	37	14	0	10.1 (± 6.9)
General public	100	55	45	9	32	22	22	15	–
Total	300	171	129	32	127	81	45	15	

As each group contained 100 participants, cell values represent both n and %

Responses to trauma-related questions relating are presented in Table 2. An OMFS was preferred by both groups to treat broken jaws, eye bone fractures, and teeth trauma. Both groups would refer to PS for facial lacerations, but a larger proportion of dentists would refer to OMFS (43% vs. 19%; p < 0.001) while a larger proportion of medical doctors would refer to general surgery (GS) (26% vs. 7%; p < 0.001). Nose fractures caused more doctors to refer to ENT (79% vs. 43%; p < 0.001) while significantly more dentists would include an OMFS referral (37% vs. 4%; p < 0.001).

**Table 2:** Trauma

Condition	Role	Plastic surgeon	Ear–nose–throat	Oral and maxillofacial surgeon	General surgeon	Others
Broken jaw	Dentist	1	0	99	0	0
	Medical Doctor	0	0	100	0	0
	*p*	1.000	–	1.000	–	–
Cut on the face (Laceration)	Dentist	50	0	43	7	0
	Medical doctor	45	0	19	26	10
	*p*	0.479	–	< 0.001	< 0.001	0.002
Eye bone fracture (orbit)	Dentist	8	4	83	5	0
	Medical doctor	3	12	67	3	15
	*p*	0.213	0.065	0.009	0.721	< 0.001
Fracture of the skull	Dentist	7	1	68	19	5
	Medical doctor	3	25	23	10	39
	*p*	0.331	< 0.001	< 0.001	0.071	< 0.001
Nose fracture	Dentist	20	42	37	1	0
	Medical doctor	17	79	4	0	0
	*p*	0.585	< 0.001	< 0.001	1.000	–
Trauma to the teeth	Dentist	1	1	72	5	21
	Medical doctor	0	0	75	0	25
	*p*	1.000	1.000	0.631	0.059	0.502

As each group contained 100 participants, cell values represent both n and %

Column p values were generated from a Chi-square test (cell count ≥ 5) or a Fisher’s exact test (cell count < 5)

Table 3 presents responses relating to pathology, showing OMFS being preferred by both groups for oral lesion biopsies, cancers of the lip, mouth, or tongue, and mouth lumps. For facial skin lesion biopsies, dentists tended to refer to OMFS (53% vs. 19%; p < 0.001), while doctors preferred PS (51% vs. 29%; p < 0.001). A patient seeking removal of a neck lump would be unlikely to get an OMFS referral from a medical doctor (7% vs. 63%; p < 0.001), who generally preferred ENT (53%; p < 0.001) or GS referrals (37%; p = 0.003). For salivary gland removal and sinus surgery, medical doctors preferred ENT (52% and 80%; p < 0.001) while dentists preferred OMFS for both procedures (90% and 51%; p < 0.001).

**Table 3:** Pathology

Condition	Role	Plastic surgeon	ear–nose–throat	Oral and maxillofacial surgeon	General surgeon	Others
Biopsy of a skin lesion on the face	Dentist	29	0	53	13	5
	Medical doctor	51	3	19	13	14
	*p*	0.001	0.246	< 0.001	1.000	0.030
Biopsy of oral lesions	Dentist	2	1	87	8	2
	Medical doctor	3	11	80	3	3
	*p*	1.000	0.005	0.182	0.213	1.000
Cancer of the lip	Dentist	10	2	87	1	0
	Medical doctor	28	11	55	3	3
	*p*	0.001	0.018	< 0.001	0.621	0.246
Cancer of the mouth or tongue	Dentist	1	1	97	0	1
	Medical doctor	0	16	84	0	0
	*p*	1.000	< 0.001	0.003	–	1.000
Lump in the mouth	Dentist	2	1	94	3	0
	Medical doctor	0	16	81	3	0
	*p*	0.497	< 0.001	0.005	1.000	–
Lump in the neck	Dentist	4	13	63	19	1
	Medical doctor	3	53	7	37	0
	*p*	1.000	< 0.001	< 0.001	0.005	1.000
Mole or lump in the face (Skin)	Dentist	41	1	39	13	6
	Medical doctor	53	0	18	12	17
	*p*	0.089	1.000	0.001	0.831	0.015
Salivary gland removal (Parotid, Submandibular)	Dentist	1	6	90	3	0
	Medical doctor	2	52	31	15	0
	*p*	1.000	< 0.001	< 0.001	0.003	–
Sinus surgery	Dentist	2	40	51	2	5
	Medical doctor	0	80	7	13	0
	*p*	0.497	< 0.001	< 0.001	0.005	0.059

As each group contained 100 participants, cell values represent both n and %

Column p values were generated from a Chi-square test (cell count ≥ 5) or Fisher’s exact test (cell count < 5)

PS or OMFS was preferred for reconstructive surgery scenarios (Table 4). Dentists preferred OMFS for children with cleft lips (p < 0.001), cleft palates (p < 0.001), and both conditions (p < 0.001), while medical doctors referred to PS most often for these procedures. Facial reconstruction following trauma saw dentists more likely to choose OMFS (69% vs. 48%; p = 0.003) and medical doctors preferring PS (52% vs. 30%; p = 0.002). Dentists referred to PS (46%) and OMFS (47%) in roughly equal numbers for facial reconstruction requiring free flaps but medical doctors were more likely to refer to PS (72%; p < 0.001) than OMFS (28%; p = 0.006). Similarly, dentists would refer to OMFS for facial bone grafts more than medical doctors (75% vs. 44%; p < 0.001). While both dentists and medical doctors would refer to OMFS for wisdom teeth removal, a large proportion of doctors would also refer to other specialties (34%; p < 0.001).

**Table 4:** Reconstructive surgery

Condition	Role	Plastic surgeon	Ear–nose–throat	Oral and maxillofacial surgeon	General surgeon	Others
Child with a cleft lip	Dentist	25	0	72	1	2
	Medical doctor	61	8	31	0	0
	*p*	< 0.001	0.007	< 0.001	1.000	0.497
Child with a cleft palate	Dentist	5	2	88	2	3
	Medical doctor	51	15	30	0	4
	*p*	< 0.001	0.002	< 0.001	0.497	1.000
Child with a cleft lip + palate	Dentist	7	3	86	1	3
	Medical doctor	60	9	31	0	0
	*p*	< 0.001	0.134	< 0.001	1.000	0.246
Dental implants	Dentist	1	1	77	5	16
	Medical doctor	0	0	80	0	20
	*p*	1.000	1.000	0.606	0.059	0.462
Facial reconstruction after facial trauma	Dentist	30	0	69	0	1
	Medical doctor	52	0	48	0	0
	*p*	0.002	–	0.003	–	1.000
Facial reconstruction with free flaps	Dentist	46	3	47	2	2
	Medical doctor	72	0	28	0	0
	*p*	< 0.001	0.246	0.006	0.497	0.497
Grafting bone in the face	Dentist	21	1	75	2	1
	Medical doctor	50	3	44	3	0
	*p*	< 0.001	0.621	< 0.001	1.000	1.000
Removal of wisdom teeth	Dentist	1	1	87	5	6
	Medical doctor	0	0	66	0	34
	*p*	1.000	1.000	< 0.001	0.059	< 0.001
Temporomandibular joint (TMJ) surgery	Dentist	1	3	95	1	0
	Medical doctor	0	10	90	0	0
	*p*	1.000	0.082	0.179	1.000	–
Taking bone from rib/hip for intra-oral grafting	Dentist	11	0	67	19	3
	Medical doctor	30	3	51	10	6
	*p*	0.001	0.246	0.021	0.071	0.498

As each group contained 100 participants, cell values represent both n and %

Column p values were generated from a Chi-square test (cell count ≥ 5) or a Fisher’s exact test (cell count < 5)

PS was preferred for all cosmetic surgeries save chin corrections, jaw deformities and discrepancies, and rhinoplasty (Table 5). OMFS was preferred for chin correction surgery by dentists more than medical doctors (73% vs. 56%; p = 0.012) and approximately equally for jaw deformities and discrepancies (93% and 92%; p = 0.788). For rhinoplasty, dentists had a stronger preference for PS (60% vs. 38%; p = 0.002) and medical doctors preferred ENT specialists (53%; p = 0.010).

**Table 5:** Cosmetic surgery

Condition	Role	Plastic surgeon	Ear–nose–throat	Oral and maxillofacial surgeon	General surgeon	Others
Chin correction surgery	Dentist	23	3	73	0	1
	Medical doctor	41	3	56	0	0
	*p*	0.006	1.000	0.012	–	1.000
Eyelid Surgery (Blepharoplasty)	Dentist	57	3	28	8	4
	Medical Doctor	58	14	4	0	24
	*p*	1.000	0.009	< 0.001	0.007	< 0.001
Face lift	Dentist	72	0	24	3	1
	Medical doctor	82	9	9	0	0
	*p*	0.093	0.003	0.004	0.246	1.000
Facial implants (silicone or other alloplasts)	Dentist	62	1	36	0	1
	Medical doctor	77	2	21	0	0
	*p*	0.021	1.000	0.019	–	1.000
Fat grafting to the face	Dentist	73	0	19	5	3
	Medical doctor	79	9	12	0	0
	*p*	0.321	0.003	0.171	0.059	0.246
Hair transplant	Dentist	65	1	3	4	27
	Medical doctor	59	9	0	3	29
	*p*	0.382	0.018	0.246	1.000	0.753
Injection of Botox and fillers	Dentist	71	3	11	4	11
	Medical doctor	76	12	0	0	12
	*p*	0.423	0.029	0.001	0.121	1.000
Jaw deformities and discrepancy	Dentist	6	1	93	0	0
	Medical doctor	8	0	92	0	0
	*p*	0.579	1.000	0.788	–	–
Laser resurfacing of facial skin	Dentist	80	0	6	1	13
	Medical doctor	75	9	2	0	14
	*p*	0.397	0.003	0.279	1.000	1.000
Problem with facial appearance or asymmetry	Dentist	54	1	44	1	0
	Medical doctor	51	3	43	0	3
	*p*	0.671	0.621	0.887	1.000	0.246
Rhinoplasty (Nose plastic surgery)	Dentist	60	35	4	1	0
	Medical doctor	38	53	9	0	0
	*p*	0.002	0.010	0.251	1.000	–

As each group contained 100 participants, cell values represent both n and %

Column p values were generated from a Chi-square test (cell count ≥ 5) or a Fisher’s exact test (cell count < 5)

**Fig. 1:**
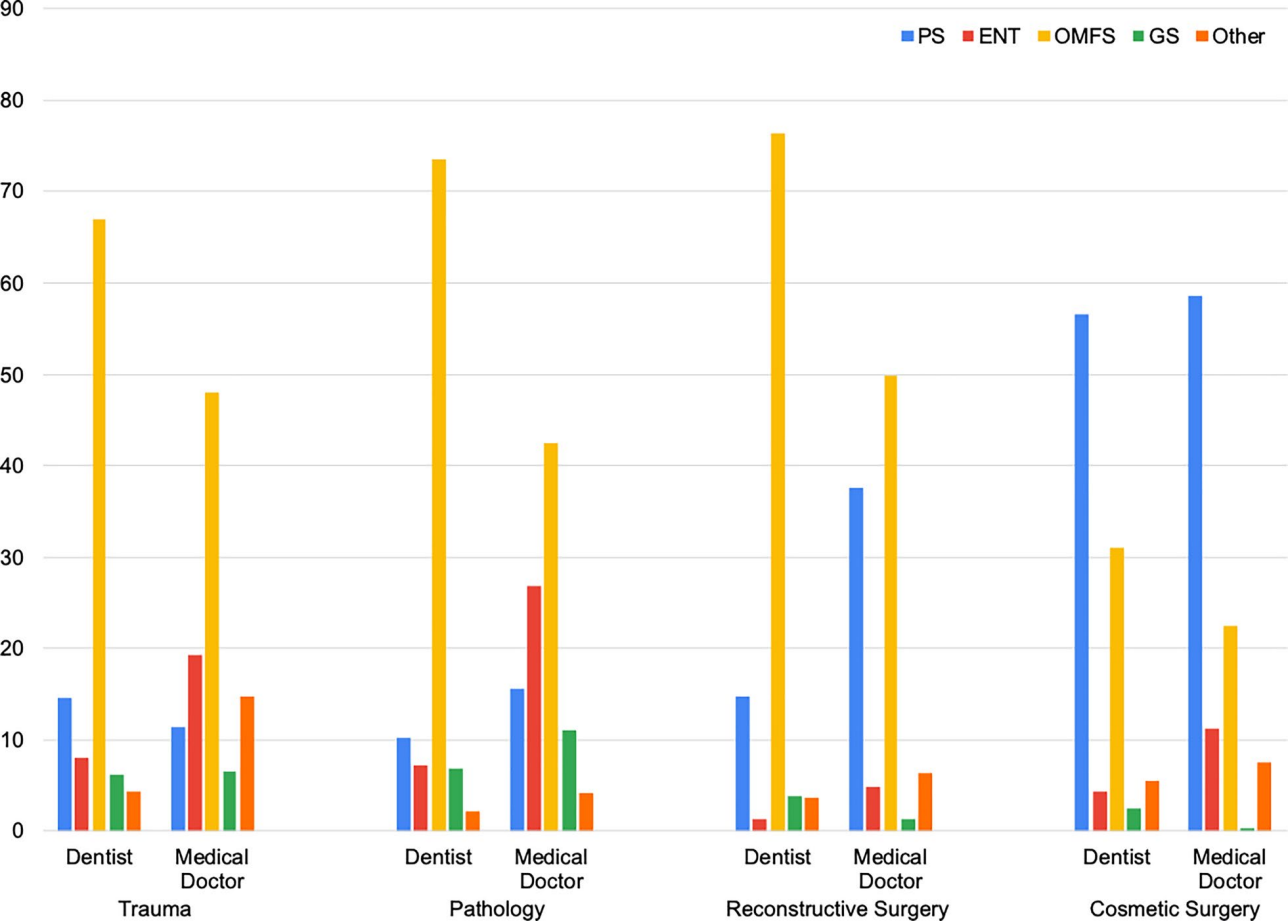
Combined responses from dentists and medical doctors in each category: trauma, pathology, reconstructive surgery, and cosmetic surgery (PS: plastic surgery, ENT: Ear-Nose-Throat, OMFS: Oral & Maxillofacial Surgery, GS: general surgery, and Other: other specialties)

Figure 1 illustrates that OMFS was preferred overall by dentists and medical doctors for most clinical scenarios in trauma (p < 0.001), pathology (p < 0.001), and reconstructive surgery (p < 0.001). PS was preferred for cosmetic surgeries (p < 0.001).

### Laypeople

One hundred laypeople were surveyed as part of this study (see Table 6), comprising 55 males and an even distribution of age ranges. Survey respondents reported their personal experiences with various specialties, with 12% having received PS treatment, 27% receiving ENT, 24% using OMFS, 77% accepting GS, and 26% getting another form of treatment.

**Table 6:** General public (laypersons)

Condition	Plastic surgeon	Ear–nose–throat	Oral and maxillofacial surgeon	General surgeon	Others
Broken jaw	0	2	92	0	6
Cut on the face (Laceration)	53	0	38	5	4
Eye bone fracture (orbit)	10	6	42	18	24
Fracture of the skull	3	1	39	30	27
Nose fracture	9	60	26	1	4
Trauma to the teeth	1	3	75	2	19
Biopsy of a skin lesion on the face	19	3	25	41	12
Biopsy of oral lesions	4	19	45	14	18
Cancer of the lip	8	7	46	18	21
Cancer of the mouth or tongue	0	17	62	4	17
Lump in the mouth	0	7	68	10	15
Lump in the neck	5	31	4	38	22
Mole or lump in the face (Skin)	58	1	20	14	7
Salivary gland removal (Parotid, Submandibular)	0	17	53	19	11
Sinus surgery	0	80	14	4	2
Child with a cleft lip	34	8	44	10	4
Child with a cleft palate	6	18	61	11	4
Child with a cleft lip + palate	9	12	70	5	4
Dental implants	1	7	62	1	29
Facial reconstruction after facial trauma	58	2	34	1	5
Facial reconstruction with free flaps	53	2	33	4	8
Grafting bone in the face	20	4	62	8	6
Removal of wisdom teeth	0	6	54	4	36
Temporomandibular Joint (TMJ) surgery	3	2	82	8	5
Taking bone from rib/hip for intra-oral grafting	13	3	58	17	9
Chin correction surgery	55	3	38	0	4
Eyelid surgery (Blepharoplasty)	53	12	19	3	13
Face lift	92	1	2	0	5
Facial implants (Silicone or other alloplasts)	79	4	12	0	5
Fat grafting to the face	88	1	6	1	4
Hair transplant	82	2	0	1	15
Injection of Botox and fillers	90	2	2	0	6
Jaw deformities and discrepancy	5	2	85	2	6
Laser resurfacing of facial skin	89	1	2	0	8
Problem with facial appearance or asymmetry	59	1	35	1	4
Rhinoplasty (Nose plastic surgery)	72	19	4	1	4

As the group contained 100 participants, cell values represent both n and %

**Fig. 2:**
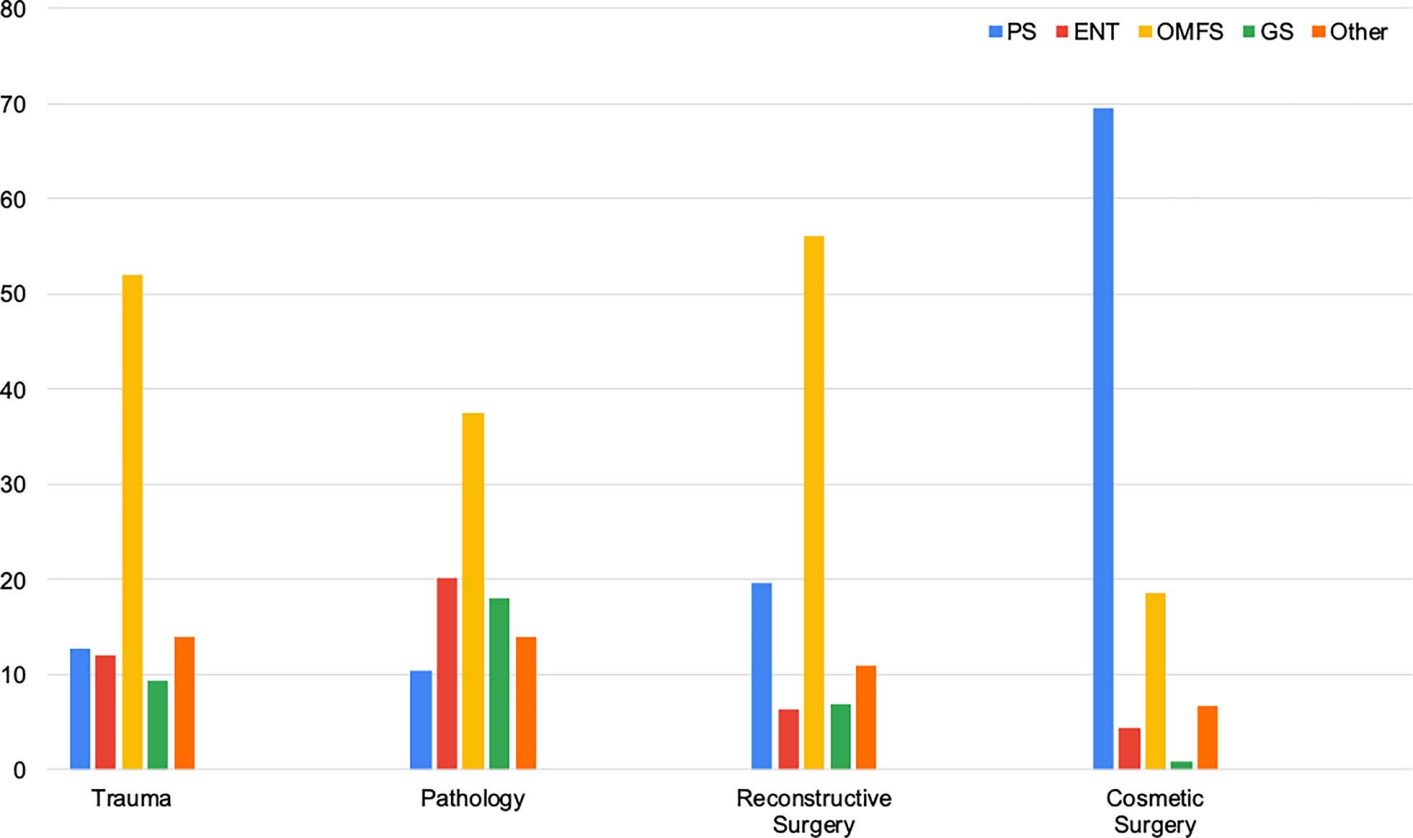
Combined responses from laypeople in each category: trauma, pathology, reconstructive surgery, and cosmetic surgery (PS: plastic surgery, ENT: Ear-Nose-Throat, OMFS: Oral & Maxillofacial Surgery, GS: general surgery, and Other: other specialties)

Overall, OMFS was preferred for issues relating to trauma, pathology, and reconstructive surgery, while PS was preferred for cosmetic surgeries (Fig. 2). Laypeople felt that ENT services were most appropriate for nose fractures and sinus surgeries. They choose PS as most appropriate for facial lacerations, moles/lumps on the face, cleft lips, facial reconstructions and all cosmetic surgeries except jaw deformities and discrepancies. GS was deemed most appropriate for facial skin lesion biopsies and neck lumps. Perception of when OMFS consultation was suitable is presented in Table 7.

**Table 7:** Perception of when to consult an oral and maxillofacial surgeon for various conditions

Condition	Dentist	Medical doctor	P^*^	Laypeople
Trauma				
Broken jaw	99	100	1.000	92
Cut on the face (Laceration)	43	19	< 0.001	38
Eye bone fracture (orbit)	83	67	0.009	42
Fracture of the skull	68	23	< 0.001	39
Nose fracture	37	4	< 0.001	26
Trauma to the teeth	72	75	0.631	75
Pathology				
Biopsy of a skin lesion on the face	53	19	< 0.001	25
Biopsy of oral lesions	87	80	0.182	45
Cancer of the lip	87	55	< 0.001	46
Cancer of the mouth or tongue	97	84	0.003	62
Lump in the mouth	94	81	0.005	68
Lump in the neck	63	7	< 0.001	4
Mole or lump in the face (Skin)	39	18	0.001	20
Salivary gland removal (Parotid, Submandibular)	90	31	< 0.001	53
Sinus surgery	51	7	< 0.001	14
Reconstructive surgery				
Child with a cleft lip	72	31	< 0.001	44
Child with a cleft palate	88	30	< 0.001	61
Child with a cleft lip + palate	86	31	< 0.001	70
Dental implants	77	80	0.606	62
Facial reconstruction after facial trauma	69	48	0.003	34
Facial reconstruction with free flaps	47	28	0.006	33
Grafting bone in the face	75	44	< 0.001	62
Removal of wisdom teeth	87	66	< 0.001	54
Temporomandibular Joint (TMJ) surgery	95	90	0.179	82
Taking bone from rib/hip for intra-oral grafting	67	51	0.021	58
Cosmetic surgery				
Chin correction surgery	73	56	0.012	38
Eyelid Surgery (Blepharoplasty)	28	4	< 0.001	19
Face lift	24	9	0.004	2
Facial implants (Silicone or other alloplasts)	36	21	0.019	12
Fat grafting to the face	19	12	0.171	6
Hair transplant	3	0	0.246	0
Injection of Botox and fillers	11	0	0.001	2
Jaw deformities and discrepancy	93	92	0.788	85
Laser resurfacing of facial skin	6	2	0.279	2
Problem with facial appearance or asymmetry	44	43	0.887	35
Rhinoplasty (nose plastic surgery)	4	9	0.251	4

^*^Statistical comparisons were performed between dentists and medical doctors only. As each group contained 100 participants, cell values represent both n and %

## Discussion

Knowledge and perception of the OMFS specialty plays a crucial role in its development. With its wide scope of practice overlapping other medical specialties, OMFS has caused a notable disparity in referring preferences among healthcare workers, students, and general public [8, 12]. Jensen mentioned that almost all medical specialties have overlapping scope to some extent, causing possible confusion when choosing an appropriate specialty for case management [14]. This necessitates establishing clear clinical guidelines and interdepartmental referral schemes in any given healthcare system. Proper referral systems ultimately lead to better patient care delivery, smaller burdens on hospital services, and greater patient satisfaction [15, 16]. Undoubtedly, OMFS surgeons will most likely continue to gather knowledge and clinical skills depending on case exposure to over time.

General public perception of OMFS is just as important health care provider perception. In Kuwait’s private sector, the public has open access to all specialty clinics. Although such unrestricted access has benefits (such as fast patient flow), they are countered by possibly inappropriate self-referrals [16]. The latter causes higher patient costs in addition to increased risk of management by health care providers not entirely skilled in a given case [16]. Our findings indicate that most medical and dental clinicians will refer to OMFS for jaw fractures, orbital fractures, and dental trauma instead of ENT, GS, or PS, which is consistent with Rocha et al.’s findings [13]. The orbital fracture findings contradict Haron et al.’s 2013 study, which stated medical doctors were less likely to refer to OMFS [7]. This may be attributed to many North American- and European-trained surgeons joining the Gulf Cooperation Council (GCC) countries workforce in recent years due to the wide implementation of the government sponsored training schemes abroad, as well as social media becoming a valid platform for patient education [7, 17].

Ameerally et al. mentioned that names given to specialties can create referral bias. (14) Our results demonstrated it is more likely for healthcare professionals and laypeople to refer cases of jaw, orbital, and dental trauma to OMFS. Such speculation is popular among other authors, such as Parnes who suggested a name change to OMFS altered its perceived spectrum. (15) However, it is extremely difficult to suggest a name that fully describes any given specialty [8].

Government-funded hospitals in Kuwait established broad guidelines on referrals [7]. Nasal fractures, for example, are to be referred to ENT specialists, which may explain why our results indicated ENT is the preferred specialty for nasal fracture management. We found that a significant number of dentists would also refer nasal fractures to OMFS. Dentists have firsthand experience with OMFS during undergraduate studies, and rotations and externships in OMFS service may grant dentists a firm understanding on the specialty’s broad scope, on the other hand, many medical doctors are unaware of OMFS training and practice [18, 19].

Regarding facial lacerations, we found that dentists and medical doctors gave equal preference to PS. However, a statistically significant difference was noted between dentists (43%) and medical doctors (19%) referring facial lacerations to OMFS. Plastic surgeons are well known for managing cases requiring special esthetic attention, which has been reported by Alnofaie et al. [8], making it unusual that GS was the second most likely specialty referral by medical doctors for facial lacerations.

Our results indicate that while OMFS is the preferred service for any pathological case of the oral cavity (Table 3), medical doctors mainly referred facial pathology requiring biopsies to PS. Our consensus among health care professionals that plastic surgeons are the most competent at treating esthetic cases is consistent with previously published studies [7, 8]. Facial lesion management is within the core of OMFS specialty with most practitioners being well trained in PS, ENT, and GS. To date, OMFS is the specialty with the greatest focus on the facial region. All surgeons are held to high standards for care delivery and esthetic outcomes. Dentists are more likely to refer any given case to OMFS save skin lumps, which tended to be referred to PS.

Sinus surgeries and salivary gland removals are within the scope of OMFS and ENT. While dentists preferred OMFS referrals, doctors preferred ENT for such case management. Haron et al.’s study found that medical doctors would refer salivary gland pathology to GS [7]. In our study, GS was the second least preferred choice of all healthcare professionals.

Regarding reconstructive surgery, dentists consistently referred all cases to OMFS first. However, dentists in Kuwait tend to refer any complicated head or neck region case to OMFS first [7]. Medical doctors mainly sent cleft lip and palate patients to PS. Children with cleft lips and palates require a team of healthcare professionals including orthodontists, pediatric physicians and dentists. The tendency of professionals to refer cleft lip and palate cases to PS could be attributed to the fact that the operatory segment of the cleft team governmental hospital established is consisted of plastic surgeons. Management of temporomandibular joints were mainly referred to OMFS by our participants, which was consistent with other published results [8, 12]. Wisdom teeth extraction and dental implants were mainly sent to OMFS. A significant number of dentists and medical doctors chose to send such cases to professionals marked as “others”, such periodontists and general dentists trained to manage minor oral surgical procedures (Table 4). Undoubtedly, their competence in such surgical intervention will reduce the burden on busy OMFS specialists in Kuwait.

For cosmetic surgery, all plastic facial procedures (except for chin corrections) were most likely to be sent to PS. Healthcare professionals prefer PS for Botox injections and hair transplants. Numerous specialties offer similar procedures, including dermatology, but the predominant worldwide perception that PS is a specialty dedicated to esthetics, with similar results found in other studies [12, 20].

Almost all facial operations should have acceptable esthetic results, rendering them cosmetic [21]. It is worrisome that healthcare professionals would refer most of such patients a specific surgical specialty, even OMFS. Specialty overlap requires consideration and referrals should be distributed equally with emphasis on any given surgeon’s expertise and skills. OMFS will evolve hugely based on experience, thus a proper referral system that considers its overlap and the training of different departments can be counted as a good investment in healthcare. Rhinoplasties are controversial as far as which specialty should offer care with PS and ENT being the top choices.

Laypeople preferred OMFS for all traumatic cases involving the face, except for nasal fractures where ENT scored higher. The Arabic name for OMFS translates literally to “jaw and facial surgery”. It seems reasonable that descriptive name and nomenclature play a key role in choosing departments. Generally, laypeople are more likely to view OMFS surgeons as performing procedures involving the head and neck region, with the only significant exception being esthetic procedures, for which they prefer PS. This public perception of PS is longstanding and affected by popular culture and social media. A major limitation in our study is that the sample size (n = 100) was small to draw a broader conclusion on a complex and diverse healthcare system in Kuwait. Given the dynamic nature of the healthcare system in Kuwait with constant influx of healthcare professionals from various global systems and training schemes, the results can show significant variations within short time frames, as seen in this study with regard to the previous views recorded in 2013.

## Conclusions

In conclusion, we observed an acceptable perception and awareness of both medical doctors and dentists towards OMFS. However, physicians seem less aware than dentists and both populations perceive cosmetic procedures as manageable by PS. We suggest developing a database of surgeons with their demonstrable surgical interventions. This database will aid referrals, since cases are sent to surgeons with the most experience, interest, or willingness to manage them. There is a need to increase awareness among all healthcare providers and the general public regarding the scope of clinical practice for the specialty of Oral and Maxillofacial Surgery, and especially towards cosmetic surgery procedures. Improving knowledge and awareness towards OMFS specialty will help in further advancing the frontiers of this specialty, and ultimately helping in delivering better care to the public.

## Data Availability

The datasets used and analyzed in the study are available from the corresponding author on reasonable request.

## Abbreviations

OMFS: Oral and maxillofacial surgery
ENT: Ear, nose, and throat
PS: Plastic surgery
GS: General surgery
